# Pullulan–dextran composite beads as bone fillers: from material design and industrial production to clinical application in oral surgery

**DOI:** 10.3389/fbioe.2026.1791131

**Published:** 2026-06-04

**Authors:** Sylvain Catros, Laurent Bidault, Sandrine Auget, Charlotte Pasquet, Camille Ehret, Rachida Aid, Mathilde Fenelon, Jean-Christophe Fricain, Joelle Amedee-Vilamitjana, Didier Letourneur

**Affiliations:** 1 Université de Bordeaux, INSERM 1026, BioTis, Bordeaux, France; 2 CHU de Bordeaux, Service de Chirurgie Orale, Bordeaux, France; 3 Siltiss, SA, Zac de la Nau, Saint-Viance, France; 4 Université Paris Cité, Université Sorbonne Paris Nord, INSERM 1148, LVTS, X Bichat Hospital, Paris, France

**Keywords:** bone tissue engineering, clinical translation, dextran, GMP, hydroxyapatite, microbeads, pullulan

## Abstract

Developing biomaterials from natural polysaccharides for medical applications presents significant challenges in terms of biocompatibility, scalability, and regulatory compliance. We report here the full development and preclinical validation of 250–300 µm pullulan–dextran–hydroxyapatite microbeads for bone regeneration, intended for use in sinus lift procedures. This class III medical device was fabricated using two pharmaceutical-grade, biodegradable, and biocompatible polysaccharides (pullulan and dextran) that were crosslinked without the use of organic solvents and embedded with hydroxyapatite microparticles. Industrial-scale production of 59 batches achieved batch-to-batch reproducibility, comprehensive physicochemical characterizations, stability, sterilization, and packaging in syringes under Good Manufacturing Practices (GMP). Preclinical validation included *in vitro* biocompatibility and toxicology testing, along with *in vivo* demonstration of efficacy in a sheep model. The injected polysaccharide beads demonstrated performance comparable to commercial clinical bovine bone granules in a two-step sinus grafting surgery in six sheep, with dental implants successfully placed 6 months post-grafting. As a novel approach, the polysaccharide beads act as a temporary scaffold that could be easily injected into the sinus cavity, gradually degrading and being replaced by dense bone tissue. Clinical trial preparation, including protocol design and regulatory files submission, culminated in regulatory approval for human use in sinus lift procedures. This work illustrates a successful translation from the laboratory to the clinic, with the journey from conceptual design to clinical trial approval of a new pullulan–dextran-based biomaterial.

## Introduction

Developing new biomaterials from polysaccharides for medical use presents important challenges (Cui et al., 2022). These hurdles span scientific, technical, regulatory, and economic dimensions. From a chemical aspect, polysaccharides derived from natural sources such as plants, algae, and microorganisms exhibit batch-to-batch variability in molecular weight, degree of branching, and purity, which complicates reproducibility (Al-Hakim, 2021). Materials derived from natural sources can raise concerns about contamination (e.g., endotoxins, heavy metals, or allergens), adding complexity to regulatory approval. Specific, robust, and accurate methods for chemical characterizations must be developed and validated. Modifying polysaccharides to achieve specific properties (e.g., biodegradability, bioactivity, and mechanical strength) without compromising their inherent biocompatibility is also complex. Achieving the necessary strength, elasticity, or toughness for specific applications (e.g., wound dressings and tissue scaffolds) requires blending polysaccharides with other materials, which can introduce major compatibility issues. Many polysaccharides are also sensitive to environmental factors such as moisture, pH, and temperature, affecting their long-term stability and usability in medical applications.

From a biological point of view, polysaccharides can trigger immune responses, especially if they are derived from non-human sources (Feng et al., 2025; Vlassopoulou et al., 2021). Ensuring low immunogenicity while maintaining bioactivity is challenging. For applications such as implants or scaffolds, achieving optimal integration with host tissues with predictable degradation rates is crucial. Evaluating the long-term behavior of polysaccharide-based materials in the body (e.g., degradation products and chronic effects) can require extensive testing. From the industrial aspect, extracting and purifying polysaccharides to meet medical-grade standards involves intricate processes that can be cost-intensive and time-consuming compared to synthetic alternatives (Simoens and Huys, 2012). Competing with existing products (synthetic polymers at lower costs) is an ongoing challenge. Ensuring uniformity in properties across production batches is challenging, given the natural origins of polysaccharides. In addition, polysaccharides often require specialized processing techniques, such as freeze-drying, electrospinning, or 3D printing (Lazaridou et al., 2022), which may not yet be well-optimized or scalable. Sterilization is also an issue that is often underestimated (Munarin et al., 2013). Addressing these hurdles requires multidisciplinary approaches, integrating advancements in chemistry, materials science, biotechnology, and engineering. Finally, polysaccharide-based materials must meet stringent regulatory requirements for medical devices.

In the field of bone regeneration, polysaccharide-based biomaterials have garnered interest as promising candidates for bone regeneration (Fricain et al., 2013; Aprile et al., 2020; Ahmed Omar et al., 2022; Lekhavadhani et al., 2025; Li et al., 2025; Du et al., 2025) due to their inherent biocompatibility, biodegradability, and their ability to mimic the extracellular matrix (Autissier et al., 2010). Our previous studies performed on a composite pullulan–dextran-based matrix, performed *in vitro* and *in vivo* using different preclinical models (Fricain et al., 2013; Fricain et al., 2018; Ahmed Omar et al., 2022), demonstrate promising results for bone tissue engineering applications. However, the clinical development of such polysaccharide-based bone filler requires a rigorous, multi-phase pathway to ensure safety, efficacy, and compliance with regulatory standards, a process that bridges fundamental material science, preclinical validation, and human trials. Letourneur et al. (2021) highlighted the role of strong clinical partnerships and close industrial collaborations to overcome barriers and regulatory hurdles for the development of new medical devices.

Bone defects arising from trauma, disease, or congenital abnormalities present significant clinical challenges, necessitating the development of advanced biomaterials that can promote regeneration while minimizing complications (Zubery et al., 2007). Traditional bone grafting approaches, such as autografts and allografts, are constrained by donor site morbidity, limited availability, variability in the processing techniques, and risks of immune rejection or disease transmission (Voss et al., 2010; Ghanaati et al., 2014; Sharifi et al., 2022). Synthetic bone fillers, including ceramics and polymers (Von Doernberg et al., 2006; Barradas et al., 2011; Bianchi et al., 2022; Ferraz, 2023; Łuczak et al., 2024; Kim et al., 2025), have emerged as alternatives, yet their clinical utility is often limited by suboptimal degradation rates or poor vascularization (Malhotra and Habibovic, 2016). The development of an innovative biodegradable bone filler composed of polysaccharides involves a systematic and multidisciplinary process analysis. The key stages of development from conceptualization to the market are tailored specifically to the unique characteristics of polysaccharide-based materials. Each stage, from design to clinical trial, presents unique challenges for polysaccharide-based materials that must be systematically addressed to ensure safety, efficacy, and regulatory compliance.

Six key steps described the conceptualization to clinical market introduction of polysaccharide-based materials in dental and orthopedic uses: 1) Conceptualization, Design and Manufacturing (batch production, sterilization, physicochemical characteristics, and risk analysis); 2) Preclinical Development (*in vitro*, *in vivo*, toxicology); 3) Regulatory Pathway and Compliance (Good Manufacturing Practices (GMP) and quality management system (QMS)); 4) Clinical Trial Preparation (protocol design, ethical, and regulatory approvals; 5) Clinical Trials (Phase I–Safety and Feasibility, Phase II–Efficacy, Phase III–Pivotal Study/Post-Trial Monitoring); 6) Regulatory Approval and Commercialization (CE mark and post-market surveillance).

Here, we describe the first four steps taken before entering clinical trials, performed in a dedicated controlled environment (quality control (QC) and Good Manufacturing Practices (GMP)) for the manufacturing, physicochemical characterizations, biocompatibility evaluation, and proof of efficacy of the injectable pullulan–dextran microbeads in the sinus lift procedure in sheep. This surgery aims to regenerate bone in the maxillary sinus area to place dental implants for teeth replacement. During a sinus lift procedure, the maxillary sinus membrane is elevated cranially to leave a space that is filled with the scaffold biomaterial. The material will act as a temporary scaffold, enabling the growth of new bone tissue (Ahmed Omar et al., 2025). Ideally, as the biomaterial degrades, it is replaced by newly formed bone, creating a robust and stable foundation for the placement of dental implants (Lundgren et al., 2017). Taken together, our positive results described here lead to the French health regulatory approval for the use of these injectable pullulan–dextran microbeads in the sinus lift procedure in humans.

## Materials and methods

### Synthesis and characterization

#### Raw materials

Sodium chloride and calcium hydroxide are from Cooperation Pharmaceutique Française (Melun-F). Phosphoric acid and sodium dodecyl sulfate are from Merck (St. Quentin-Fallavier), and sodium hydroxide and ammonia are from MERCK KGaA (Darmstadt, Germany). Refined rapeseed oil 8002-13-9 is from Olvea Vegetable Oils (St Leonard-F), and sodium trimetaphosphate (Na_3_P_3_O_9_)> 95% is from Molekula Group (Darlington, United Kingdom). The literature review performed on these raw materials did not evidence specific toxicological concerns.

#### Polysaccharides

Pullulan consists of glucose units linked through α-1,4 and α-1,6 glycosidic bonds. Pullulan was manufactured by fermenting a non-GM strain of *Aureobasidium pullulans* (*A. pullulans*) with starch syrup as a substrate. Pullulan 9057-02-7 was obtained from Nagase (Düsseldorf, Germany). The internal analysis for batch 7F0621 of pullulan had a viscosity of 166 mm^2^ s^−1^, a bulk density of 0.259 g/cm^3^, 0.03% sulfated ash, 2.6% loss on drying, and heavy metals such as Pb < 5 ppm. The microbial contamination (TAMC) was <1 CFU/g.

Dextran is a polymer of D-glucose units connected by α-1,6 linkages derived by fractionation of the dextrans produced by fermentation of sucrose (food grade, from sugar beets) using *Leuconostoc mesenteroide*s strain (NCTC 10817). Clinical grade dextran 9004-54-0 was purchased from Pharmacosmos (Holbaek, Denmark) or from Sigma Aldrich 31,392 (Saint-Quentin-Fallavier, F) for R&D analysis. The internal analysis for batch DM1081 of clinical-grade dextran found 0.3% sulfated ash and 4.1% loss on drying (<7 for specifications). The quantity of bacterial endotoxins was 0.8 UI/g (<16 for specifications). The microbial contamination (TAMC) was <1 CFU/g (<100 for specifications).

The molecular weight of the polysaccharides was determined by HPLC-SEC with aquagel columns from Agilent and eluted in NaNO_3_ 0.3 M, pH = 7. The pullulan was from four different batches of Nagase (#1I1321: 207 840; #4B18: 210 660; #2J1721: 216 005; #IG0659006B: 211 137). The dextran was from three different batches of two manufacturers (Pharmacosmos batch DK424#1: 178 900; Pharmacosmos batch DK424#2: 180 300; Sigma batch 1335895: 198 980).

#### Preparation of micro-hydroxyapatite

Micro-hydroxyapatite was produced by a crystallization process starting from calcium hydroxide, phosphoric acid, ammonia, and water at room temperature, as previously described (Fricain et al., 2013). All raw materials used in the synthesis of micro-hydroxyapatite suspension were pharmaceutical grade and had a synthetic origin. The process resulted in a suspension in water of 2.8% (w/w) of hydroxyapatite microcrystals evaluated with the particle size analyzer, Mastersizer 3000 (Malvern Panalytical, Palaiseau, France). Using dynamic light scattering (DLS) by Nanosizer-Malvern, no nanoparticles were detected in these suspensions (data not shown). Transmission electron microscopic images also confirmed the presence of agglomerates of micro-sized aggregates of HA > 100 nm, even after sonication (data not shown). The suspension was decontaminated by autoclaving.

#### Preparation of microbeads

Microbeads were prepared using a home-made static mixer with a helicoidal unit by the dissolution of pullulan (75% w/w) and dextran (25% w/w) powders into hydroxyapatite aqueous suspension, crosslinked with STMP in NaOH (1 M final), and extruded through a nozzle to fall into the oil bath. Experimentally, Glycobone® microbeads are produced by the controlled mixing of two aqueous feed streams, Feeder A and Feeder B, at a 1:1 volumetric ratio using a dual-pump system. Each feeder is prepared separately, and gelation is initiated immediately upon their combination. Feeders A and B are formulated by dissolving pullulan and dextran powders into an aqueous suspension containing 2.8% (w/w) hydroxyapatite (HA), achieving final polymer concentrations of 13.2% (w/w) pullulan and 4.4% (w/w) dextran. To initiate crosslinking, sodium hydroxide (NaOH, 10 M) is added to Feeder A to reach a final concentration of 2 M, such that after mixing with Feeder B, the NaOH concentration is 1 M. Concurrently, sodium trimetaphosphate (STMP, 22.5% w/v in water) is added to Feeder B to achieve a final concentration of 4.5% w/v, yielding a post-mixing concentration of 2.25% w/v. Both feed solutions are homogenized, and entrapped air bubbles are removed through three successive vacuum cycles (30 s under vacuum followed by 2 min at atmospheric pressure per cycle). Following preparation, the two feed streams are combined using a static mixer equipped with a helical mixing element. The resulting homogeneous mixture is then extruded through a nozzle into an oil bath, where it is emulsified under high shear (450 RPM) generated by an anchor impeller. This emulsification process produces calibrated microbeads that are allowed to crosslink for 1 hour. All solution preparation steps are conducted in an ISO 7 classified environment. Extrusion and mixing operations are performed within a closed pilot unit, also maintained under ISO 7 conditions.

The ratio of pullulan to dextran was previously optimized without any HA in relation to the biocompatibility and degradability of the resulting hydrogels (Abed et al., 2008; Abed et al., 2011). With this optimum polysaccharide ratio, the hydroxyapatite content was then also optimized based on the homogenous distribution of the HA particles within the matrix and to the *in vivo* performance on ectopic bone formation (Ehret et al., 2017).

The microbeads were first sieved on a 1 mm sieve to eliminate the excess of oversized objects. Beads were then transferred into the rinsing pilot unit, where they were subject to a continuous flow of fresh buffer. Beads were kept inside the pilot unit by a 100 µm sieve. The rinsing step was carried out in a closed pilot unit. The sieving of the microbeads of Glycobone® allowed for the separation of the oversized beads. Three sieves were used: 1 mm, 0.5 mm, and 0.3 mm. Beads retained on the 0.3 mm sieve were freeze-dried (Cryotec, Lunel, France).

The final product was packaged in pre-filled syringes with 0.32 g of freeze-dried beads. Syringes were packaged in a blister. The process was carried out in an ISO5 environment. Syringes were then sent to Top Clean Packaging (TCP) (Peschadoires, France) for the final packaging. Glycobone® was supplied as a pre-filled 3 mL syringe from Medmix (Haag, Switzerland) containing the freeze-dried powder of polysaccharide beads to be reconstituted with an injectable saline solution. The syringes were presented in a double blister, together with a cannula to be used in the ejection of the reconstituted and rehydrated beads.

Glycobone® was sterilized by gamma irradiation at 25±3 kGy by Ionisos (Dagneux, France), a company certified under GMP and ISO 13485. The gamma irradiators, powered by cobalt-60 (emitting photons with energy levels of 1.17 MeV and 1.33 MeV), effectively eliminate microorganisms throughout the product and its packaging with minimal temperature elevation and no chemical residues. The radiation has a penetrating power of up to 1 m. An automated system feeds the products into the irradiation cell, passes them in front of the radiation source according to a defined circuit and controlled speed to ensure a uniform dose, and then removes them. To comply with loading/palletizing plans validated during the quality assurance process, these palletizing protocols were meticulously followed. Dosimetric release allows for the immediate processing, verification, and release of products for shipment.

Three irradiation doses (25 kGy ±10%, 35 kGy ±10%, and 40 kGy ±10%) were tested on non-conditioned syringes. Post-irradiation, the products were analyzed by quality control to assess the impact on physicochemical properties. The 40 kGy dose was identified as the maximum acceptable dose as it induced only slight effects. Consequently, Glycobone® was sterilized at the validated dose of 25 ± 2.5 kGy.

#### Characterizations of Glycobone® microbeads

A total of 59 batches of Glycobone® beads were produced over the 4–5 years. During this time period, the methods were progressively developed and qualified. The final released batches indicated as “Conform” complied with the following criteria.

##### HA content

The hydroxyapatite content in Glycobone® was determined by the calcium concentration obtained by ICP/OES (Thermo Scientific, iCAP PRO XP Duo + SDX HPLD). The hydroxyapatite content is expressed in % w/w and corresponds to a 100% crystalline hydroxyapatite phase with the formula: Ca_10_(PO_4_)_6_(OH)_2_. The correlation coefficient of the calibration line was r ≥ 0.995 for the 396.85 nm Ca wavelength. For three sample preparations: % relative standard deviation (RSD) ≤5.0%.

##### Water content

The water content in Glycobone® was determined by coulometric titration on samples immediately after the freeze-drying step. As the lyophilized powder is insoluble in solvent, the coulometer (Karl Fischer 917 from Metrohm, Villebon-sur-Yvette, F) is equipped with an oven to extract water from the beads (150 °C; Gas flow: 90 mL/min). The measurements were performed by weighing 20 mg of standards (n = 3) and samples (n = 3) in each vial. The recovery versus standard was 97.5%–102.5%.

##### Size of microbeads

The particle size was determined by laser diffraction according to the European Pharmacopeia 2.9.31 using a particle size analyzer (Mastersizer 3000) in dry state and hydrated conditions with NaCl 0.9% (w/v) solution. The following values were used: refractive index (1.538), absorption index (0.01), and particle density (1.00 g/cm^3^). The values of 3 g samples of Dx10, Dx50, and Dx90 as percentiles were collected and averaged with three measurements for each sample.

##### Swelling ratio

The analytical method used for the Glycobone® beads (50 ± 5 mg) swelling ratio in NaCl 0.9% were validated by a gravimetric method as the ratio of mass of hydrated/freeze-dried beads after 1 hour. The mass of freeze-dried products must be corrected for the water content. The precision of the method was characterized by a maximum RSD of 2.2% for repeatability (≤ 3.0%).

##### Phosphorus assay

The determination of total phosphorus content in Glycobone® is based on a complexometric method. Phosphorus reacts with molybdate in an acidic medium to form a phosphomolybdate complex, which, in turn, reacts with vanadium to create a yellow coloration. This yellow complex is proportional to the concentration of total phosphorus in the sample and can thus be quantified by visible spectrophotometry at 405 nm. The method used is as follows: exactly 50 mg ± 5 mg of freeze-dried beads and 1.0 mL of 10% HNO_3_ solution were added to a 2-mL Eppendorf microtube. The microtubes were closed and incubated for 2 h at 70 °C in an oven. For each sample (n = 3), 50 μL of the mineralized beads solution, 2.0 mL solution A (250 mg of ammonium monovanadate with 40 mL of distilled water, 10 mL of 65% nitric acid), and 2.0 mL solution B (5.0 g of ammonium molybdate tetrahydrate into a 100 mL water) was used, and then the volume was made up with 3% HNO_3_. A calibration curve was performed for each batch using phosphoric acid. Samples were read at 405 nm with a spectrophotometer.

##### Residual oil determination

The presence of oil residue in Glycobone® was monitored by three analytical methods. 1) Middle-infrared spectroscopy (MIR): The characteristic peak of the oil in a middle-infrared measurement is pointed at ∼ 1740 cm^−1^. 2) Confocal laser scanning microscopy: Lipids are visualized in green due to the dye BODIPY® (Thermo Fisher Scientific, Ref D3922). 3) Limit test by gas chromatography with downstream flame ionization detector (GC/FID): Residual rapeseed oil in Glycobone® is extracted from Glycobone® by diethyl ether. The fatty acids are derivatized into methyl esters to enable their detection in GC-FID (SHIMADZU CQ-CPG-002 with an Agilent Column HP-INNOWAX (Ref 19091-N-113I: 30 m × 0.25 µm x 0.32 mm). The prepared samples are compared with samples spiked with Colza oil.

##### Enzymatic degradation

The *in vitro* Glycobone® degradation is based on an *in vitro* degradation system with two enzymes: pullulanase and dextranase. The purpose was not to mimic the *in vivo* conditions but to have a reliable, reproducible method to follow in the synthesis of Glycobone®. The beads (200 mg), rehydrated in 20 mL of PBS 1X, are immersed in 10 mL of an enzyme solution containing both pullulanase at a final enzymatic activity of 18.0 U/g, and dextranase at a final enzymatic activity of 147.5 U/g (Sigma Aldrich, Ref E2412 and Ref D0443, respectively). The enzymatic activities are assayed monthly using the enzymatic kit from Megazyme. These include the Limit-Dextrizyme method (Ref T-LDZ 03/15) and the assay of 1,6-alpha-dextranase using alpha-dextrazyme tablets (Ref T-DEXT 10/19). Every 10 min up to 80 min, the remaining mass of Glycobone® beads is weighed. This yields the degradation kinetics (% degradation versus time). The enzymatic degradation was performed on three replicates from 12 batches, repeated every 3 days. Values of the enzymatic degradation assay were considered independent and ensured that the product met the desired quality, efficacy, and safety levels. The specifications are set to the minimum and the maximum values, with the mean values obtained from the 108 degradation time points. The t_50%_ was calculated (in min) to obtain the time for 50% degradation by weight, as the direct intercept with the Y value at 50%.

##### Endotoxins

The determination of the specification for endotoxin content in Glycobone® is based on the European Pharmacopeia 5.1.10: “Guidelines for using the test for bacterial endotoxins.” For the calculation, Glycobone® is considered to be administered intravenously for a dose corresponding to two syringes of 0.32 g. According to these calculations, the endotoxin specification in Glycobone® is set to: ≤7 UI/g.

Batches were subjected to endotoxin assays using the limulus amebocyte lysate (LAL) method and/or in-house testing using the recombinant Factor C (r-FC) assay. LAL assays were subcontracted to three different companies: Icare (63360 Saint-Beauzire, France), ACM Pharma (45270 Bellegarde, France), and Eurofins Amatsi Analytics (04310 Peyruis, France).

##### Sterility

The sterility test was performed by Icare (St Beauzire, France) according to ISO 11737-2 Sterilization of healthcare products — Microbiological methods — Part 2: Tests of sterility performed in the definition, validation, and maintenance of a sterilization process. The total aerobic microbial count (TAMC) and the total combined yeasts and molds count (TYMC) in CFU/g were calculated according to European Pharmacopeia 2.2.12.

##### Photosensitivity and shelf life

The photosensitivity of the Glycobone® device conditioned in the commercial packaging was studied according to the ICH Q1B (International Council for Harmonization of Technical Requirements for Pharmaceuticals for Human Use) guideline. The stability study was performed on a technical batch under two stability ICH conditions: 18 months of storage under long-term conditions (i.e., 25 °C/60% RH) and after 6 months under accelerated conditions (i.e., 40 °C/75% RH).

### Biocompatibility of Glycobone®

A biocompatibility study was performed by Icare (St Beauzire, France), according to ISO 10993-1:2018 (cytotoxicity testing, intradermal tolerance, skin sensitization, acute systemic toxicity, pyrogen testing, mutagenicity/genotoxicity, and a 1-month implantation toxicity study).

Glycobone® was tested for cytotoxic effect by direct contact based on the requirements of ISO 10993-5:2009 Biological evaluation of medical devices — Tests for *in vitro* cytotoxicity. A study of extractables and leachables was then performed by Rescoll (Pessac, France) in ultra-pure water and in hexane solvent on the biocompatibility batch. The extract obtained from Glycobone® was assessed for cytotoxicity according to ISO 10993-5:2009 Biological evaluation of medical devices — Tests for *in vitro* cytotoxicity. The sensitizing assay is based on the requirements of ISO 10993-10:2010 Biological evaluation of medical devices — Tests for irritation and skin sensitization. Five male guinea pigs were used for the control groups for both the polar and non-polar extracts. Ten animals each were used for the two extracts of Glycobone®. The irritation test was performed according to ISO 10993-10:2010 Biological evaluation of medical devices — Tests for irritation and skin sensitization on three male albino New Zealand rabbits with intracutaneous injection. Acute systemic toxicity was based on the requirements of ISO 10993-17:2017 Biological evaluation of medical devices — Tests for systemic toxicity. Material-mediated pyrogenicity was tested in the framework of ISO 10993-11:2017 Biological evaluation of medical devices — Tests for systemic toxicity. Mutagenic assay was based on the requirements of ISO 10993-3:2014 Biological evaluation of medical devices — Tests for genotoxicity, carcinogenicity and reproductive toxicity. The clastogenic assay was performed according to ISO 10993-3:2014, Biological evaluation of medical devices — Tests for genotoxicity, carcinogenicity and reproductive toxicity, and the mutagenicity assay was based on the requirements of ISO 10993-3:2014 Biological evaluation of medical devices — Tests for genotoxicity, carcinogenicity and reproductive toxicity.

Glycobone® was also implanted into the subcutaneous tissues of female Wistar rats for 1 month and 3 months according to ISO 10993-6:2016 Biological evaluation of medical devices — Test for local effects after implantation. Glycobone®, 0.32 g per animal, that is, the human dose, was implanted in five (for 1 month) or 10 (for 3 months) female rats at 0.16 g per site with two sites per animal. Other groups of five or 10 female rats received two sham implantations under the same conditions. Body weight evolution, biochemical, enzymology, and hematology parameters, macroscopic examination of the main organs and organ weight, and histopathological examinations were performed.

### Proof of concept of the efficacy of Glycobone® in large animals

#### Experimental model and surgical procedure

Animal experiments were performed in accordance with the “Principles of Laboratory Animal Care” recommended by the National Society for Biomedical Research in France. Surgery was carried out in an accredited animal facility (NAMSA, Chasse sur Rhône, France, agreement number 3808710001), after obtaining a positive evaluation of the animal experiment from the Ethic Committee of the French Ministry of Research (N°23,109–2019112114593417V3 and 15,043–2018050710524583V4). Conventional instruments of oral surgery were used, including a surgical rotary handpiece and a surgical burr. The bilateral sinus lift procedure was performed on six adult sheep (mean weight = 70 kg), aged 3 years old.

The protocol used for general anesthesia was the following: induction was done using an intravenous injection of diazepam 0.3 mg/kg (Valium®). Butorphanol 0.2 mg/kg (Torphasol®) and propofol 2–5 mg/kg (Propovet®) were used to maintain the anesthesia.

After skin shaving and disinfection (povidone iodine), a skin incision was performed, and superficial muscles were dissected to access the lateral aspect of the maxillary sinus. Then, a “bone window” was created in the maxilla using a burr mounted on a handpiece under saline irrigation (NaCl 0.9%). The sinus membrane was carefully lifted in the cranial direction with dedicated instruments. Glycobone® and positive control (Bio-Oss®, bovine bone granules from Geistlich Pharma AG, Switzerland) materials were allocated randomly to be injected into one side of each animal. These animals received bilateral sinus grafts: one test and one control. The volume of graft material (test and control) used to fill each sinus lift model was standardized using the 5 mL syringes prepared with both materials (Glycobone® and the commercial control, Bio-Oss®), both rehydrated in buffered saline. Resorbable sutures (Vicryl 4.0, Ethicon®) were used to close the muscle layer, and surgical staples were used to close the superficial skin layer. Post-operative follow-up was performed every day for 2 weeks by a veterinary surgeon from NAMSA to evaluate the aspects of the wounds and the general condition of the animals. Oxyterin® spray was applied to the wounds every other day for 1 week. The animals received intramuscular (IM) injections post-surgery of analgesics (buprenorphine: Buprecare® 0.01 mg/kg twice a day for 3 days), antibiotics (amoxicillin: Duphamox LA® 15 mg/kg IM every other day for 10 days post-surgery, and non-steroidal anti-inflammatory drugs (flunixin: Meflosyl® 1 mg/kg, every day for 5 days).

Six months (M6) after the filling of the sinus cavity with Glycobone® or the control Bio-Oss®, another surgical procedure was performed under general anesthesia to place a titanium dental implant (Anthogyr) through the zygomatic bone. After a 3-month healing period for osseointegration of the dental implants, the animals were euthanized using a 1 mL/5 kg intravenous pentobarbital injection, euthanized 9 months (M9) after implantation of the Glycobone® (n = 6) and control Bio-Oss® (n = 6). A block resection of the sinus was done for all samples. The blocks were fixed into 10% neutral buffered formalin for 3 days and submitted to NAMSA for micro-computed tomography, histomorphometry, and histopathological analyses, conducted in compliance with Good Laboratory Practice (GLP) requirements. The scheme of implantation of the injectable materials and the dental implant is depicted in Supplementary Figure S1A. The different analyzed areas and the regions of interest (ROI) around and at a distance from the dental implant for histology and histomorphometry analysis are presented in Supplementary Figure S1B. Intraosseous implantation of Glycobone® or the control Bio-Oss® in the femoral condyle was performed on explanted samples of bone (femoral condyle) for further micro-CT analysis to obtain an image for the time point T0 (less than 6 h of implantation).

#### Micro-CT analysis

After complete fixation in 10% NBF, all samples were imaged with a cone beam micro-computed tomography (µCT 40, SCANCO, Switzerland). A transversal scan was performed. The micro-CT specimen holder was sized at 37 mm diameter × 75 mm length. The measured data were filtered using a Gaussian filter with finite filter support and filter width. The images were then segmented to separate the material from the tissue background. A 3D reconstruction was performed to obtain 3D images. For quantitative analysis, a standardized volume of interest (VOI) was defined as a parallelepiped (8 mm × 7 mm × 6 mm depth) placed between the endosteal side of the cortical bone and the bevel of the dental implant, as illustrated in Supplementary Figure S1C. The following measurements were made within the volume of interest (VOI) for all samples:

- BV + MatV: Bone + material volume within the VOI (mm^3^)
- (BV + MatV)/TV: Bone + material volume density within the VOI to total volume (%)
- BIC: Bone-to-implant contact (%): that is, the percentage of the dental implant perimeter in direct contact with the newly formed mineralized bone tissue.

#### Histological analysis and histopathological evaluation

The samples were dehydrated in alcohol solutions of increasing concentrations, cleared in xylene, and embedded in polymethylmethacrylate (PMMA). For each implantation site, one frontal central longitudinal section of the dental implant was performed in Area 1, and a second central longitudinal section was performed in Area 2, parallel to the first one (Supplementary Figure S1B). Sections of approximately 40 µm thickness were obtained by the microcutting and grinding technique (Exakt™ system). Sections were stained with a modified Paragon for qualitative, semi-quantitative, and histomorphometric analyses.

Qualitative and semi-quantitative histopathological evaluations of the local tissue effects at the implantation sites were conducted in agreement with the ISO 10993. The histological parameters evaluated were scored as described in Supplementary Table S1. The peri-implant bone growth was also qualitatively and semi-quantitatively evaluated.

#### Histomorphometry evaluation

The histomorphometry analyses were conducted on 24 sections from Area 1 and 24 sections from Area 2. Sections were then scanned (Zeiss AXIOSCAN Z1, x20) and analyzed with a color image analyzing system CaloPix (Tribun, France) to perform a semi-automatic analysis.

For Area 1, two standardized regions of interest (ROI a and ROI b) were defined as rectangles placed at the subcortical bone, on each side of the implant, as shown in Supplementary Figure S1B. For Area 2: one standardized region of interest (ROI c, Supplementary Figure S1B) was defined as a rectangle placed under the cortical bone, at a distance from the dental implant. The quantitative analysis was performed to assess the percentage of the following parameters within the ROIs: i) Total mineralized area (TMA), that is, the total area of the newly formed bone and bone substitute in the ROI a, b, and c.; ii) Bone to implant contact (BIC), that is, the percentage of the dental implant perimeter in direct contact with the mineralized bone tissue; iii) Fibrous tissue density, that is, the ratio of the area of fibrous tissue in the ROI a, b, and ROI c.

### Statistical analysis

Data for local tissue effects and performance (bone content) were expressed as mean +standard deviation without statistical comparison (only six sheep were used). Qualitative and semi-quantitative histopathologic evaluations of the local tissue effects at the implantation sites were conducted according to the standard (ISO 10993-Part 6). Statistical analysis (5% risk) was conducted for the micro-CT and histomorphometric parameters with statistical software (Software SPSS, SPSS. Inc.). Comparison between groups was performed by ANOVA (micro-CT analysis and histomorphometric analysis).

### Submission to regulatory authorities for a clinical trial

All documents were submitted to legal authorities in France, CPP (IRB-Institutional Review Board), and ANSM (French National Agency for the Safety of Medicines and Health Products). The clinical protocol on 40 patients was designed for a longitudinal analysis (9 months for each patient), open-label study to evaluate Glycobone®, as a bone filling material in a lateral sinus lift procedure.

## Results

### Preparation of pullulan–dextran–HA microbeads

**FIGURE 1:**
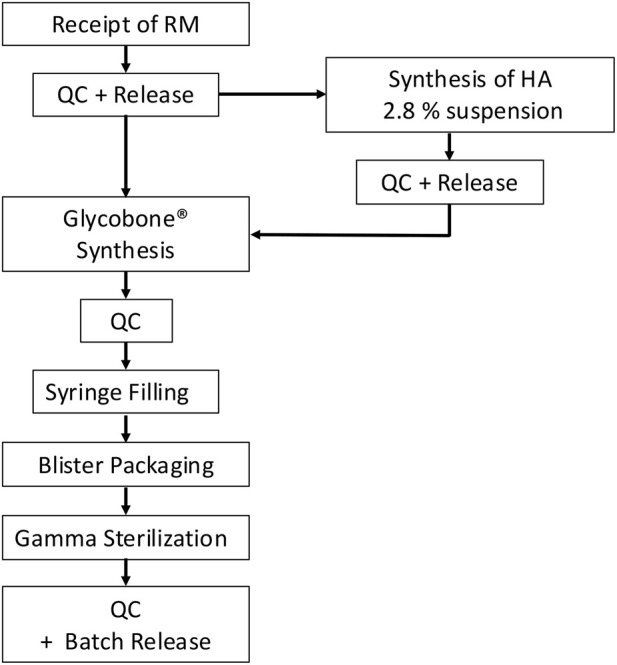
General scheme for the whole production process. The first step is the receipt of raw materials (RMs), followed by the quality control (QC). A suspension of hydroxyapatite (HA) was used for the synthesis of pullulan–dextran–HA microbeads (Glycobone®). After QC, the product was loaded into dedicated syringes, packaged, and sent for gamma irradiation. The final sterilized products were then ultimately controlled for quality and released. A total of 59 distinct batches were produced over a 4–5 year development period. The objective was to scale up the production process from 1 g to approximately 60 g of freeze-dried beads per batch in a reproducible manner and within an ISO 7 to ISO 5 cleanroom environment. Each synthesis requires approximately 6 days to produce, which includes 2 days dedicated to the freeze-drying step.

We have designed a glycomaterial, Glycobone®, composed of pullulan and dextran crosslinked by phosphate bonds, containing dispersed non-stoichiometric hydroxyapatite microcrystals. Whereas the synthesis has been largely described at the lab scale (Fricain et al., 2018; Lanouar et al., 2018; Grenier et al., 2019; Grenier et al., 2022), we specifically adapted here the methods at an industrial scale to produce about 60 g of freeze-dried beads per batch in an environmentally controlled manufacturing unit (ISO 7 to ISO 5 cleanroom environment). Each room and microbiological security area was controlled under ISO 14644. In this article, we presented all processes from raw materials through detailed characterizations (quality control) to the final batch release (Figure 1). A total of 59 different batches were produced.

The first step for the synthesis of Glycobone® was dedicated to the synthesis of micro-hydroxyapatite, which is a raw material produced internally. X-ray diffraction (XRD) analysis gave mean values of Ca/P ratio of 1.804, crystallinity of 84%, and the calculated CaO mass fraction of 4.406%. The Ca/P ratio of pure stoichiometric mineral (hydroxyapatite) is 1.67. The size and Ca/P ratio of hydroxyapatite are not independent properties but work in concert to determine its biological outcome.

The sizes of HA obtained from 24 different batches of micro-hydroxyapatite are reported in Table 1. The values ranged from 3 µm to 40 µm. We evidenced i) low variation in size from batch to batch and ii) the absence of nanosized hydroxyapatite materials, further confirmed by Nanosizer measurements.

**TABLE 1:** Size measurements of micro-hydroxyapatite. The values were obtained with a Mastersizer from 24 different batches of micro-hydroxyapatite. Nanosizer measurements also confirmed the absence of nanosized hydroxyapatite materials. Values are means, standard deviation (SD), and relative SD, with min and max data. Dx10, Dx50, and Dx90 are percentile values. These are statistical parameters obtained directly from the cumulative particle size distribution. They indicated the size below which 10%, 50%, or 90% of all particles are found. Dx50 is thus the average particle size of micro-hydroxyapatite.

Size of micro-HA	Dx10 (µm)	Dx50 (µm)	Dx90 (µm)
Mean	3.88	11.25	30.9
Standard deviation (SD)	0.43	1.24	3.79
Relative SD %	11.1	11.05	12.3
Min	3.8	9.45	24.8
Max	4.87	14.2	37.6

The second step in the preparation of Glycobone® was dedicated to the preparation of microbeads made of crosslinked pullulan, dextran and micro-hydroxyapatite. Glycobone® is composed of two high molecular weight polysaccharides, pullulan and dextran. The peak molecular weight for pullulan was 211 ± 3 kDa from four different batches, and the peak molecular weight for dextran was 186 ± 11 kDa from three different batches from two manufacturers.

**FIGURE 2:**
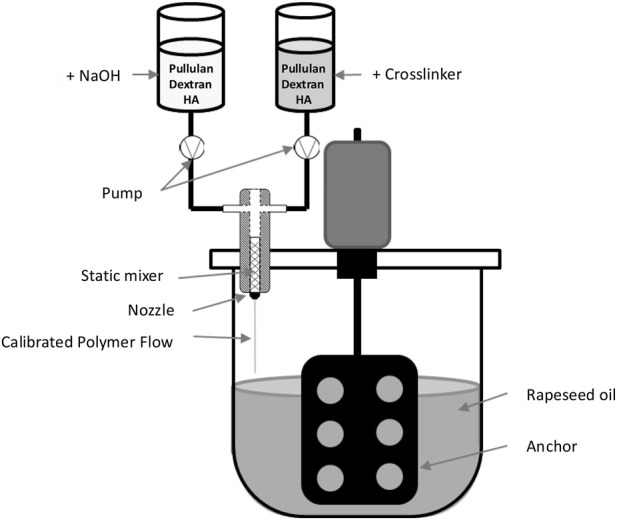
Scheme for the preparation of microbeads. They are produced by the controlled mixing of two feeders at a 1:1 volumetric ratio using a dual-pump system. Each feeder is prepared separately, and gelation is initiated immediately upon their combination. Both feeders are first formulated by dissolving pullulan and dextran powders into an aqueous suspension containing hydroxyapatite (HA). Sodium hydroxide (activator) is added to one feeder, whereas sodium trimetaphosphate (crosslinker, STMP) is added to the other feeder. Following preparation, the two feed streams are combined using a static mixer equipped with a helical mixing element. The resulting solution extruded using a calibrated nozzle is then poured into an oil bath.

The polysaccharides in powder were directly solubilized in the aqueous hydroxyapatite suspension. The increase in the pH by the addition of NaOH (1 M final) allowed the transformation of alcohol groups of the polysaccharides into alcoholate groups. The addition of the crosslinker, sodium trimetaphosphate (STMP), chemically crosslinks alcoholate functions by phosphoester bonds (Lack et al., 2007; Grenier et al., 2022). In the industrial process, two tanks were first filled with pullulan. Dextran powders were dissolved in an aqueous suspension of HA. NaOH was added to one tank, and STMP was added to the other. The two feed streams were then combined with a dual pump through a static mixer equipped with a helical mixing element. During the gelation process, the solution was poured into oil at high speed to form microsized polysaccharide beads (Figure 2). The crosslinked beads were washed in a proprietary rinsing pilot unit afterward to remove residuals (i.e., oil, NaOH, salts). Diameters of the obtained microspheres were separated by sieving. Beads between the sieves of 0.5 mm and 0.3 mm were freeze-dried.

### Characterizations of pullulan–dextran–HA beads

#### Physico-chemical characteristics

The hydroxyapatite content in Glycobone® was determined with calcium content by ICP/OES using a 100% crystalline hydroxyapatite phase using the formula Ca_10_(PO_4_)_6_(OH)_2_. By assuming an equal reactivity for pullulan and dextran chains, the final chemical composition of Glycobone® was thus calculated to be (w/w): pullulan (68.9%), dextran (22.9%), and micro-HA (8.5%).

Results of the water content obtained from 16 batches of freeze-dried Glycobone® yielded a mean value of 2.9% ± 0.49 (%RSD = 16.8%). The maximum water content to release the batches was thus set at <5% (w/w).

The mean sizes from 22 batches of polysaccharide beads in the dry state are reported in Table 2. As the Glycobone® medical device is reconstituted in physiological serum before implantation, the swelling ratio of the beads was studied in 0.9% NaCl. Values in hydrated conditions are also reported in Table 2. Beads around 250 µm in dry and 300 µm in hydrated conditions were thus obtained.

**TABLE 2:** Size measurements of microbeads of Glycobone^®^. The values of size measurements in µm were obtained with a Mastersizer from 22 different batches of dry or hydrated Glycobone^®^ in 0.9% NaCl. Values are means, SD, and relative SD. Data for Dx10, Dx50, and Dx90 are percentile values from the cumulative bead size distribution. They indicate the size below which 10%, 50%, or 90% of all beads are found. Dx50 is thus the mean size in µm of Glycobone^®^ beads.

Beads of Glycobone®	Dry state			Hydrated in 0.9% NaCl		
​	Dx10	Dx50	Dx90	Dx10	Dx50	Dx90
Mean (n = 22) in µm	97	242	466	153	306	511
Standard deviation (SD) in µm	24	25	39	40	53	93
Relative SD %	24	11	8	26	17	18

The swelling ratio was measured by weight between dry beads and beads rehydrated in 0.9% NaCl. Results of the swelling ratio obtained from 10 different pilot batches of Glycobone® reconstituted in NaCl 0.9% gave a mean value of 8.9 ± 0.56 (% RSD 6.2%). The specifications for Glycobone® produced at the pilot scale were thus set at 8.0–10.0.

The determination of phosphorus content in Glycobone® is based on a phosphomolybdate complex, and the analytical method has been validated over the range from 0.0 to 9.0 μg/mL corresponding to 0.60 to 3.6% w/w of Glycobone® with a correlation coefficient r ≥ 0.995. The precision of the method was characterized by a maximum RSD of 4.7% for repeatability (≤ 5.0%). The specifications for Glycobone® produced at the pilot scale were fixed at 25.8–39.2 μM/g. The content of phosphorus is attributed to both phosphate bonds due to the crosslinking by STMP (Lack et al., 2007) and the presence of hydroxyapatite.

The absence of oil residue in Glycobone® resulting from the precipitation of beads during the crosslinking step was monitored by three analytical methods (Supplementary Figure S2): middle-infrared spectroscopy (MIR) at ∼ 1740 cm^−1^, confocal laser scanning microscopy, where lipids are visualized with BODIPY®, and GC/FID with specifications <0.5% w/w.

To obtain a master curve for degradation, the *in vitro* enzymatic degradation was performed from 12 batches every 10 min to 80 min on three replicates, each repeated on three consecutive days. A total of 108 values of the enzymatic degradation were considered to fix the specifications with minimal and maximal values. A reference curve with a biphasic shape with mean, min, and max values every 10 min for 80 min was thus obtained with a mean t_50%_ at 32 min.

A typical result of analysis before gamma irradiation for the batch (#190160) used for large animal experiments (see below) yielded the following characteristics: swelling ratio in 0.9% NaCl of 8.6; size (µm): Dx10, 62.3 ± 0.7; Dx50, 204 ± 3; and Dx90, and 408 ± 6; phosphorus content, 31.2 µM; degradation t_50%_ = 27 min; and absence of oil evaluated by infra-red, microscopic examination, and CG-FID.

### Sterilization

Glycobone® was sterilized by gamma irradiation at 25 kGy. The absence of microbiological growth was demonstrated over a period of 24 months for the long-term condition (25 °C/60% RH) and 6 months for the accelerated condition (40 °C/75% RH). From batches irradiated with gamma rays at 25 kGy, a study of extractables and leachables in saline solution for 72 h at 37 °C demonstrated no volatile, semi-volatile, or non-volatile organic compounds (within the detection limits of gas chromatography coupled to mass spectrometry).

The result of analysis obtained for the gamma-irradiated batch (#190172) used for large animal experiments (see below): swelling ratio of 10.5; size in 0.9% NaCl (µm), Dx10: 50.5± 2; Dx50, 174 ± 2; and Dx90, 386 ± 7; phosphorus content, 26.8 µM; degradation t_50%_ = 21 min; and absence of oil.

### Reconstitution of beads for preclinical and clinical experiments

The sterilized beads in the syringes could be reconstituted with a sterile saline solution (0.9% sodium chloride). Approximately 5 mL of saline solution is required for the reconstruction within less than 1 min of one syringe of 0.32 g of dry beads (Supplementary Material Video S1). The movie reported here displayed the reconstitution of Glycobone® and its injection via a cannula.

#### Photosensitivity and shelf life

Light illumination did not affect the visual aspect and the physico-chemical characteristics of the Glycobone® device. The Glycobone® kit device conditioned in the commercial packaging did not require storage protected from light (Supplementary Figure S3). No significant change was observed in the final sterilized product characteristics after 18 months of storage under long-term conditions (25 °C/60% RH) and after 6 months under accelerated conditions (40 °C/75% RH). Moreover, the sealing integrity was stated as conforming after 18 months of storage at ambient temperature and not protected from light. A provisional shelf life of 18 months has been proposed.

### Biocompatibility

A biocompatibility study was performed for cytotoxicity test, intradermal tolerance, skin sensitization, acute systemic toxicity, pyrogen test, mutagenicity/genotoxicity, and implantation toxicity study (1 month and 3 months).

#### Cytotoxicity assay

*In vitro* assays with trypan blue exclusion test (three replicates per sample) demonstrated that Glycobone® is not cytotoxic in saline extracts (0.32 g in 2 mL) nor in direct contact with cells for 24 h. For indirect contact, values are 95 ± 9% for 100% extract, 95 ± 3% for 50%, 96 ± 3% for 10%, 105 ± 7% for 1% extract, versus 102 ± 2% for negative control culture media and 51 ± 7% for positive control (phenol 0.64 mg/mL). For direct contact, values are 95 ± 3% versus 107 ± 8% for negative control culture media and 52±3% for positive control (latex).

#### Sensitizing tests on albino Guinea pigs

Glycobone® was tested in the extract prepared using a polar solvent and a non-polar solvent on 30 guinea pigs initially exposed to Glycobone® by intradermal injections and epidermal application (induction exposure). The extent and degree of skin reaction to the exposure in the test animals were comparable to those of control animals that underwent sham treatment during induction and received the same challenge exposure. No macroscopic cutaneous reactions attributable to allergy were recorded during the examination following the removal of the occlusive dressing (challenge phase) from the treated animals. No cutaneous intolerance reaction was recorded in animals from the control group. Consequently, Glycobone® does not need to be classified as a skin sensitizer.

#### Pyrogenic tests

Glycobone® was injected into three rabbits by the intravenous route. The individual maximal temperature increase 3 h post-injection was 0.40 °C. The total maximal temperature increase was 1.05 °C (lower than the limit of 1.15 °C). Consequently, the pyrogenic test demonstrates that Glycobone® does not need to be classified as a pyrogenic medical device.

#### Irritation tests in the rabbit following intracutaneous injection

The polar and non-polar extracts at 0.2 g/mL of Glycobone® were prepared using physiological saline and cottonseed oil, respectively. No cutaneous reaction was noted on the areas treated with the non-polar solvent at 24 h, 48 h, and 72 h after the injections. No cutaneous reaction was noted on the areas treated with the polar extract 24 h, 48 h, and 72 h after the injections. A very slight to well-defined erythema was noted on the areas with the non-polar extract 14 h, 48 h, and 72 h after the injection. This reaction was associated with very slight edema, 24 h and 48 h after the injection in one animal. The primary irritation index (P.I.I) is 0.00 with the polar extract and 0.18 with the non-polar extract. Based on these results, the polar and non-polar extracts of Glycobone® meet the requirements of the test (primary irritation index <1.0).

#### Acute toxicity tests

The polar extracts of Glycobone® at a ratio of 0.2 g/mL were administered by intravenous route at a dose of 50 mL/kg body weight to a group of five mice. The non-polar extracts of Glycobone® were administered by intraperitoneal route at a dose of 50 mL/kg body weight to a group of five mice. No mortality occurred during the study. No clinical signs related to the administration of the polar and non-polar test extracts were observed. The body weight evolution of the animals remained normal throughout the study for both the treated and control animals. The LD_50_ value of the polar extract of the item Glycobone® was >50 mL/kg by intravenous route. The LD_50_ of the non-polar extract of Glycobone® was >50 mL/kg by the intraperitoneal route.

#### Local effect and systemic toxicity: subcutaneous implantation in rats for 1 month

Glycobone® was implanted in five rats at a dose of 0.32 g per animal (2 × 0.16 g), and a group of five rats received sham implantations under the same conditions. No mortality occurred during the 1-month experimental period. The body weight evolution of animals from the Glycobone® group was similar to the body weight evolution of the animals from the control group (p > 0.05). No effect of the treatment with Glycobone® was noted on biochemical, enzymatic, or hematological parameters. The median values were similar (p > 0.05) between the sham and Glycobone®-treated groups for plasmatic white blood cells count, red blood cells count, hemoglobin level, blood clotting time (Quick time), hematocrit, total and differential leukocyte count (neutrophils, eosinophils, basophils, lymphocytes, and monocytes), platelet count, and erythrocytes indices (MCV, MCH, and MCHC). The macroscopic examination of the animals at the end of the study did not reveal changes in the main organs or at the implantation sites of Glycobone® and the control groups. No macroscopic anomalies were seen on lymph nodes draining the implantation sites (axillary lymph nodes). No effect of the treatment was noted in the organ weight of the animals. There was no evidence of tissue necrosis nor fibrous encapsulation. Glycobone® implanted into the subcutaneous tissues of rats for 1 month did not induce evident signs of systemic toxicity. The histopathological examinations confirmed that the implantation of Glycobone® did not induce material-related microscopic findings in organs distant from the injection sites.

A slight to severe edema was noted from day 2 in all sites implanted with Glycobone® (10/10) and was still observed in eight implantation sites (8/10) at the end of the implantation period (very slight to slight intensity). The histopathological examinations showed that the subcutaneous implantation of Glycobone® in rats for 1 month elicited a minimal to mild inflammatory reaction at implantation sites. There was no evidence of tissue necrosis or fibrous encapsulation. When compared to sham-operated sites, Glycobone® was considered to be slightly irritating at the used dose. It is noted that a supra-physiological dose of Glycobone® was implanted (for a human sinus lift application, 300 mg of material was injected in the bone cavity, whereas 2 × 160 mg were injected in each rat).

#### Local effect and systemic toxicity: Subcutaneous implantation in rats for 3 months

Glycobone® implanted into the subcutaneous tissues of 10 rats for 3 months did not induce evident signs of systemic toxicity. The histopathological examinations confirmed that the implantation did not induce any related microscopic findings in organs distant from the injection sites. A very slight to moderate edema was noted in all sites implanted with the test item between day 2 and day 17 (20/20) and was still observed at the end of the experimental period (day 91) in 15 sites (15/20, very slight to moderate intensity). When compared to sham-operated sites, Glycobone® was considered to be slightly irritating. Again, a supra-physiological dose of Glycobone® was implanted in rats subcutaneously (300 mg in humans vs. 2 × 160 mg in each rat). The histopathological examinations showed that the subcutaneous implantation of Glycobone® in rats for 3 months elicited a minimal inflammatory reaction at implantation sites. There was no evidence of tissue necrosis or fibrous encapsulation.

*In vivo* studies also demonstrated that Glycobone® is not considered clastogenic, does not induce any mutagenic change, and does not induce a mutagenic effect.

All together, these results evidenced that Glycobone® was not cytotoxic by direct or indirect contact, not sensitizing, not irritating, not mutagenic, not clastogenic, did not show signs of acute systemic toxicity, and did not show signs of material-mediated pyrogenicity. The evaluations of endpoints for irritation and sensitization potential, material-mediated pyrogenicity, genotoxicity, and carcinogenicity potential are thus strongly in favor of the biocompatibility of the medical device Glycobone®.

### Large animal efficacy

We evaluated the performance and local tissue effects of the bone substitute developed (Glycobone®) in a sheep maxillary sinus bone augmentation model. The main parameters evaluated were the *in vivo* biocompatibility, inflammation, bone volume augmentation in the sinus, and dental implant osteointegration. Sheep received a sinus lift bone augmentation using the Glycobone® or a commercial bovine bone xenograft (positive control: Bio-Oss®). A “two-step surgery” was performed as the dental implants were inserted 6 months after sinus grafting of the two biomaterials. The samples were analyzed 9 months after the sinus lift (Supplementary Figure S1).

#### Micro-CT analysis

**FIGURE 3:**
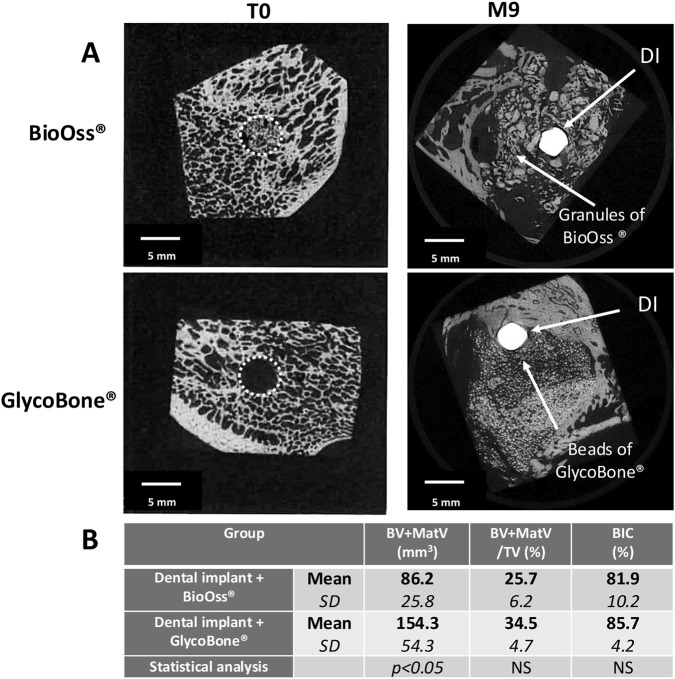
**(A)** Representative images of the two biomaterials implanted (Bio-Oss® and Glycobone®) following their implantation (T0) or after 9 months (M9). **(B)** Quantitative analysis of the bone parameters: BV + MatV: bone volume + material volume within the VOI (mm^3^) around the implant as defined in Supplementary Figure S1C; BV + MatV/TV: bone volume + material volume density within the VOI to total volume in percentage (%); BIC: bone + material to implant contact in percentage (%). NS, no significant difference; SD, standard deviation; significant difference *p* < 0.05 (n = 6).

The summary of the micro-CT analysis is reported in Figure 3, and representative micro-CT pictures obtained at T0 following implantation in bone tissue and after 9 months are shown in Figure 3A. At T0 (Figure 3A), the Glycobone® was radiolucent, while the positive control biomaterial Bio-Oss® (xenograft) was radiopaque at T0. The mineral granules of Bio-Oss® were clearly identifiable at this time point.

After 9 months (M9) of implantation, it was still possible to distinguish the granules of Bio-Oss®. Interestingly, while the Glycobone® was radiolucent at T0, the microbeads of Glycobone® were morphologically identifiable because of their mineralization at this time of implantation (Figure 3A). Regarding the micro-CT quantification performed around the dental implant as described in Supplementary Figure S1C, no significant difference was observed between the Glycobone® and Bio-Oss® samples for BV + MatV/TV nor BIC parameters (Figure 3B). Interestingly, the BV + MatV remains significantly higher for Glycobone® than Bio-Oss® samples (Figure 3B).

#### Histological analysis

Histological analyses were performed in the two areas depicted in Supplementary Figure S1B, around the dental implant (Area 1) and at a distance from the implant (Area 2).

**FIGURE 4:**
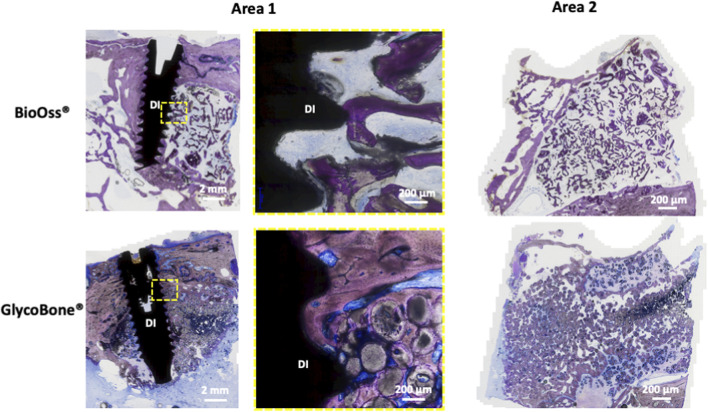
Histological analysis of the sections of the two biomaterials (Bio-Oss® and Glycobone®) 9 months post-implantation with the dental implant (DI), stained with a modified Paragon. Representative photomicrographs of both groups in the two areas (Area 1 and Area 2) (defined as described in Supplementary Figure S1B). Area 1: high-magnification view corresponds to the yellow dotted box on the images.

In Area 1, for the control group (Bio-Oss®), the inflammatory reaction observed around the biomaterial was minimal and characterized by a slight infiltration of macrophages, giant cells/osteoclasts, and rare lymphocytes. This positive control was moderately to markedly osteointegrated and appeared slightly degraded. The new bone formation, supported by the osteoconductive material Bio-Oss®, was graded “marked.” On average, the bone augmentation reached or exceeded the dental implant length with a low intra- and inter-site heterogeneity. The bone remodeling was advanced. A moderate grade of fibrous tissue remained present in the treated sites. The dental implant was osteointegrated and was associated with signs of osteoconduction (Figure 4).

In Area 1, the inflammatory reaction observed for Glycobone® around the beads was minimal and characterized by a moderate infiltration of macrophages and giant cells/osteoclasts with a few lymphocytes.

**FIGURE 5:**
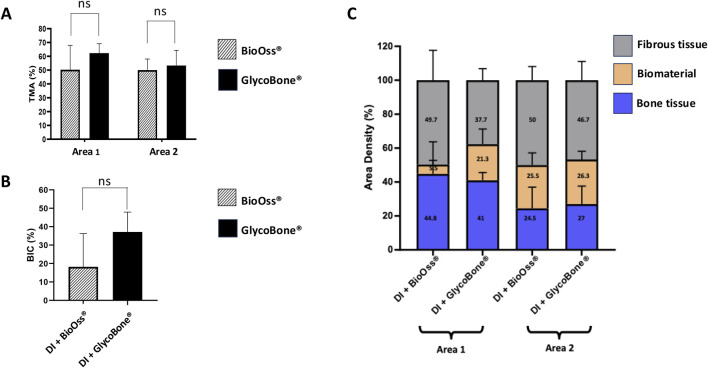
Histomorphometry quantitative analysis. **(A)** Total mineralized area (TMA) in percentage (%) in the two areas (Area 1 and Area 2) (as defined in Supplementary Figure S1B) of the tissue formed by the two groups of biomaterials (Bio-Oss® and Glycobone®) 9 months post-implantation (including 3 months with a dental implant (DI)). ns, not significant (n = 6). **(B)** Cumulative density parameters of the fibrous tissue density, biomaterial density, and bone density, in percentage (%), in the two areas (Area 1 and Area 2). No significant differences were observed between the groups for any component. **(C)** Bone-implant contact (BIC) density in percentage (%) quantified in Area 1 in contact with the dental implant. ns, no significant differences were observed between materials (n = 5 for Bio-Oss® and n = 6 for Glycobone®).

Glycobone® was markedly osteointegrated and appeared slightly to moderately degraded. The new bone formation, supported by the osteoconductive Glycobone® material, was graded “marked.” On average, the bone augmentation reached or exceeded the dental implant length with a low intra- and inter-site heterogeneity. The number of microbeads of Glycobone® observable after 9 months appeared higher than in the control Bio-Oss® group. The bone remodeling was of a moderate grade. A moderate grade of fibrous tissue remained present in the treated sites. The dental implant was markedly osteointegrated and was associated with marked signs of osteoconduction (Figure 5).

In Area 2, around the dental implant, the osteointegration of granules was marked in the Glycobone® group and slightly lower in the control Bio-Oss® group (moderate to marked), while the bone augmentation was marked and similar in the two groups. In the sinus cavity, marked evidence of osteoconduction associated with the implanted biomaterials was similarly observed in the two groups. The density appeared higher in the Glycobone® group than in the control Bio-Oss® group in the dental or sinus cavity. Slightly higher signs of article degradation were noted in the Glycobone® group. Finally, at a distance from the dental implant, the cortical bone closure was slightly more advanced in the Glycobone® group (Figure 4).

#### Histomorphometric analysis

Semi-quantitative histological analysis quantification was performed for each area around (Area 1) and at a distance from the dental implant (Area 2) for the inflammatory parameters (polymorphonuclear cells, lymphocytes, macrophages, and giant cells/osteoclastic cells) (Supplementary Table S1A), as well as for other tissue response parameters, including bone tissue parameters dedicated to this study (osteoblastic cells, osteoconduction, osteointegration, and bone remodeling) (Supplementary Table S1B).

Glycobone® and Bio-Oss® have similar values of total mineralized area (TMA) generated around the dental implant (Area 1) or at a distance within the sinus cavity (Area 2) (Figure 5A), confirming the micro-CT results (Figure 3A). No significant differences were observed between the Glycobone® and Bio-Oss® groups around the dental implant (Area 1) or at a distance within the sinus cavity (Area 2) for the bone tissue, biomaterial presence, or fibrous tissue area density (Figure 5B). At the dental implant area, although a higher bone-implant contact (BIC) value was observed for the Glycobone® group (37.1 ± 10.8%; n = 5) than the Bio-Oss® group (18.2 ± 18%; n = 6), this difference did not reach statistical significance (Figure 5C). Altogether, our results evidence that Glycobone® performed equivalently to the clinical reference material Bio-Oss®.

### Submission to regulatory authorities for a clinical trial

The industrial production with qualified products and procedures allowed proposing the polysaccharide-based material as a medical device for a clinical trial. We also obtained evidence reported here that the structure of Glycobone® with optimal hydration and mechanical properties was favorable for easy injection for bone defect filling, to support new bone tissue formation. Its progressive degradation into safe degradation products (glucose) ensured tissue remodeling and maturation of new bone to support dental implant osseointegration. We have thus prepared all documents for submission to legal authorities in France, the CPP (IRB-Institutional Review Board) Ethics Committee for study protocols on population and the ANSM (French National Agency for the Safety of Medicines and Health Products).

We have submitted the clinical protocol for a longitudinal, open-label study to evaluate Glycobone® as a bone filling material in a lateral sinus lift procedure in 40 patients. We obtained the agreement from CPP in December 2021 and from ANSM on 11 January 2022 (Supplementary Figure S4). Two subsequent amendments were then required to adapt to changes in the fabrication methods and in raw materials, including the new low-endotoxin pullulan from Hayashibara. The respective agreements were obtained in 2022 and 2023.

## Discussion

The successful development of new polysaccharides for medical devices represents a significant milestone in advancing biomaterials science, offering innovative solutions for a wide range of medical applications (Li et al., 2025). Polysaccharides, natural polymers derived from renewable sources such as plants, algae, and microorganisms, possess unique properties, such as biocompatibility, biodegradability, and the ability to be chemically modified. These attributes make them highly attractive for developing medical devices that interact harmoniously with biological systems. Materials such as chitosan, alginate, and hyaluronic acid have been successfully engineered into hydrogels, films, and scaffolds for various therapeutic and surgical applications (Yadav et al., 2022; Cui et al., 2024; Wang et al., 2025). Polysaccharides are used in drug delivery systems (Nimbalkar et al., 2023; Hsu et al., 2024; Tu et al., 2025) to provide targeted and sustained release of pharmaceuticals, minimizing side effects and enhancing therapeutic outcomes (Forero Ramirez et al., 2020).

Polysaccharide scaffolds have gained recognition in tissue engineering, where their structural similarity to extracellular matrix components supports cell adhesion, proliferation, and tissue regeneration (Silva et al., 2015; Alizadeh et al., 2024). Among them, pullulan and dextran are promising biomaterials for tissue repair (Singh et al., 2016; Banerjee et al., 2021). The regulatory approval of several polysaccharide-based medical devices further underscores the feasibility of these materials for clinical use. For instance, hyaluronic acid-based products have been successfully commercialized for joint lubrication, dermal fillers, and wound care, while alginate-derived materials have found use in dental applications and wound dressings (Priya et al., 2024). Moreover, the natural abundance and sustainability of polysaccharides align with the global push for greener and more environmentally friendly materials. Their ability to be sourced from renewable resources, coupled with advancements in manufacturing processes, has contributed to their growing acceptance in the medical field.

Our academic research, through *in vitro* and *in vivo* studies that have been the subject of numerous publications, has been the driving force behind the development of this tissue engineering product and its transfer to the clinical setting, thanks to the dedicated controlled environment and the use of quality control (QC) and Good Manufacturing Practices (GMP). The results demonstrated the successful development of Glycobone® for the first four steps of the roadmap: 1) manufacturing and characterization, 2) preclinical development, 3) regulatory pathway and compliance, and 4) clinical trial approval.

The composite polysaccharide material used was engineered from pullulan and dextran to meet the physicochemical and biological demands of bone repair. Dextran and pullulan are biodegradable and biocompatible natural polysaccharides already used as pharmaceutical ingredients. Glycobone® is a glycomaterial composed of pullulan and dextran crosslinked by phosphate bonds containing dispersed non-stoichiometric hydroxyapatite microcrystals. STMP is a common crosslinking agent currently used in the food industry as a non-toxic cyclic triphosphate crosslinker. It does not have any adverse effects on humans and is often used for the preparation of crosslinked hydrogels and microspheres for pharmaceutical applications (Dulong et al., 2004). The synthesis has been extensively described at the laboratory scale (Fricain et al., 2018; Lanouar et al., 2018; Grenier et al., 2019; Grenier et al., 2022). All processes from the receipt of raw materials to the batch release were specifically adapted for this study at an industrial scale and manufactured in a cleanroom environment. A total of 59 different batches were produced, and reproducibility has been extensively studied at all steps.

The hydroxyapatite particles used to promote the osteoinductive and osteoconductive properties of the composite matrices were produced at room temperature to preserve their bioactivity (Schlaubitz et al., 2014; Fricain et al., 2018). They were previously characterized by transmission electron microscopy, showing numerous aggregates of particles, always larger than 1 µm and smaller than 100 µm. The size of HA ensures high bioactivity, efficient protein adsorption, and a topography that stimulates osteogenesis, while the non-stoichiometric Ca/P ratio ensures a controlled and timely biodegradation profile that matches the body’s natural healing process. For a bone filler material, such as the pullulan–dextran–hydroxyapatite microbeads described, incorporating HA with these biomimetic characteristics-microscale with high surface area and a bone-like Ca/P- ratio is a strategic choice to create a temporary scaffold that promotes rapid integration and is progressively replaced by dense, functional host bone. The homogenous distribution of these aggregates within the composite matrix was also a key parameter for providing osteoconductive properties and improving mechanical properties (Wang, 1998). From a regulatory standpoint, the absence of nano-objects in the polysaccharide-based composite matrix does not hinder regulatory procedures (Malik and Patil, 2020).

Detailed physicochemical and biological characterizations were carried out. The results on the raw materials and the resulting crosslinked polysaccharide microbeads produced at an industrial scale under GMP conditions are reported here. Glycobone® is a medical device with no pharmacological components in the product. In addition, Glycobone® does not have any animal products or organic solvents. In the framework of Regulation 2017/745 on medical devices (Annex VIII), Glycobone® is a class III medical device based on classification rule 8. In the framework of ISO 10993-1:2018, Glycobone® is classified as an implant medical device in long-term contact (>30 days) with tissue/bone.

The biological risk analysis investigated the overall biological evaluation of the device: the materials; intended additives, process contaminants, and residues; packaging materials that contact the device can transfer chemicals to the device and then indirectly to the patient or clinician; leachable substances; degradation products; other components and their interactions in the final product; the performance and characteristics of the final product; physical characteristics of the final product. The biocompatibility analyses performed according to ISO 10993-5:2009 (cytotoxicity), ISO 10993-10:2010 (sensitizing assay), ISO 10993-17:2017 (acute systemic toxicity, material-mediated pyrogenicity), and ISO 10993-3:2014 (mutagenic assay) complete our results obtained *in vitro* on the absence of cytotoxicity.

Here, we have selected microbeads of 250 µm–300 µm for the production of Glycobone®. The rationale and advantages of controlling the microbead size concern several properties: i) their injectability and handling; ii) the space maintenance and their mechanical stability, and finally, iii) their surgical versatility. Microbeads in this size range, when suspended in blood or in saline, create a homogenous, paste-like consistency that can be easily extruded through a cannula, as shown in Supplementary Material Video S1. Smaller microbeads could form a denser, clay-like paste that is difficult to inject, while larger beads could cause clogging. In addition, the paste formed by these beads is cohesive, meaning it tends to remain a unit when implanted. This prevents the graft material from migrating or dispersing into surrounding soft tissues after injection, a critical feature for a precise procedure like a sinus lift. Moreover, the packed bed of 250–300 μm microbeads provides immediate structural support in the defect site. This property, known as space maintenance, is essential, ensuring a defined volume is preserved for new bone to form. While not load-bearing like cortical bone, it provides sufficient mechanical stability for the initial healing phase in a protected site like a sinus lift. At last, this size is versatile. It can be used in a variety of bone defect shapes and sizes, as the injectable paste can conform to complex geometries, ensuring intimate contact with the host bone walls, which is crucial for osteoconduction.

The preclinical development described in this study is based on previous preclinical data from tests performed on small animals. Here, a clinical model in oral surgery has been proposed to develop a new competitive medical device of natural origin, with no animal-derived components. The sinus lift procedure was selected for the proof of concept of the efficacy of the microbeads of Glycobone®.

In this study, we provided evidence in a large-animal model (sheep) that the tested composite polysaccharide (pullulan–dextran–micro-hydroxyapatite) was at least as efficient as the reference clinical material (Bio-Oss® made of bovine bone granules). The bone regeneration obtained after a 6-month healing period allowed dental implant placement and its osteointegration (observed on radiological and histological analyses) (Figures 3–5). A significant advantage for clinicians is the ability to monitor the progression of the mineralized tissue around the implant with Glycobone® microbeads using radiographic techniques (such as cone beam computed tomography), compared to Ca-P-based materials, such as Bio-Oss®, which are always visible upon implantation, making radiographic monitoring less straightforward. The longitudinal monitoring will also allow evaluating the performance in terms of mineralization (Figure 3), and the direct contact with the dental implant, once implanted, quantified here by micro-CT analysis (Figure 3B). In addition, to improve and reduce the time of these two-step surgical procedures, the follow-up of the mineralized tissue can also predict the time required for the implantation of the dental implant, generally performed from 3 months after the filling with the bone substitute. The presence of remaining material after 9 months remains comparable between the two groups (Bio-Oss®, Glycobone®) (Figure 5C). Moreover, the amount of fibrous tissue appears to be higher for Bio-Oss® (49.7%), compared to microbeads of Glycobone® (37.7%; Figure 5C) in Area 1, close to the dental implant.

The animal studies using pullulan–dextran–microhydroxyapatite also provided satisfactory results for the absence of potential systemic toxicity or local adverse reactions, forming the foundation for regulatory submissions to initiate human trials. All these results validate Glycobone® as a promising glycomaterial for bone reconstruction in the posterior maxilla. The complete administrative dossier containing the product description with detailed production methods and characteristics, together with the Investigative Brochure containing the clinical protocol, was thus submitted and approved by regulatory authorities.

Some specific challenges with the use of polysaccharides can be outlined here. 1) Biodegradation balance: tailor the degradation profile to align with the bone healing timeline, ensuring material clearance without compromising structural support. 2) Batch-to-batch consistency: address variability in polysaccharide sources and processing to ensure consistent device performance. 3) Sustainability and scalability: implement environmentally sustainable practices for polysaccharide sourcing and production. 4) Regulatory hurdles: navigate through regulatory requirements, particularly for polysaccharide-based biomaterials. 5) Cost-effectiveness: balance manufacturing costs with the affordability of the device for widespread clinical use. Overcoming these hurdles is critical to translating innovative polysaccharide-based solutions into safe, effective, and clinically relevant bone regeneration therapies.

We have thus addressed the first four steps from conceptual design to clinical trial approval: 1) Manufacturing (batch production, sterilization, and physicochemical characteristics); 2) preclinical development (*In vitro*, *in vivo*, and toxicology); 3) regulatory pathway (GMP and quality management system); and 4) clinical trial preparation (Protocol, ethical, and regulatory approvals). Future plans require more investments in time and money to launch clinical trials (Phase I/II—safety, feasibility, and efficacy; Phase III—Pivotal Study/Post-Trial Monitoring), to obtain regulatory approval (CE mark and post-market surveillance), and to start commercialization. We envision that at least five more years of intensive work and an investment exceeding €10 million will be required to complete the remaining steps toward clinical translation. This period will also necessitate expanding our current manufacturing capabilities. Building on the promising initial results with Glycobone®, we plan to conduct additional clinical trials to broaden its clinical applications. These will include studies focused on maxillary bone repair using guided bone regeneration techniques (Aprile et al., 2020; Ahmed et al., 2023) as well as orthopedic reconstructions (Fricain et al., 2013), with the ultimate goal of extending the product’s clinical use.

## Conclusion

The relatively limited number of new polysaccharides for medical devices reflects the interplay of complex scientific, technical, and economic challenges. Although these barriers are significant, they also represent opportunities for innovation and collaboration. By addressing these limitations, the field of biopolymers can advance toward the development of safer, more effective, and sustainable polysaccharide-based medical devices. Thus, the development of a polysaccharide-based bone filler represents a convergence of material innovation, biological understanding, and clinical pragmatism. We have provided an example of the main steps carried out from design to approval for clinical trial of a new pullulan–dextran material as a class III medical device in the context of bone regeneration. The journey from conceptual design to clinical trial approval of this pullulan–dextran-based bone filler illustrates both the challenges and opportunities inherent in developing polysaccharide-based medical devices. By adhering to a structured pathway from benchtop design to market, this material holds the potential to address needs in orthopedics and maxillofacial surgery, offering a safe, sustainable, and efficient solution for bone regeneration. To help researchers and developers better anticipate the necessary resources (human, financial, and external) and timelines, the roadmap illustrated here should facilitate the translation of promising glycomaterials into safe and effective clinical solutions.

## Data Availability

The raw data supporting the conclusions of this article will be made available by the authors, without undue reservation.
