# Supplementary material for: Pullulan–dextran composite beads as bone fillers: from material design and industrial production to clinical application in oral surgery
Source: Front Bioeng Biotechnol. 2026 Jun 4;14:1791131. doi: 10.3389/fbioe.2026.1791131 (PMC13276405; doi:10.3389/fbioe.2026.1791131)
Supplement: Supplementary file 1 [file DataSheet2.pdf]

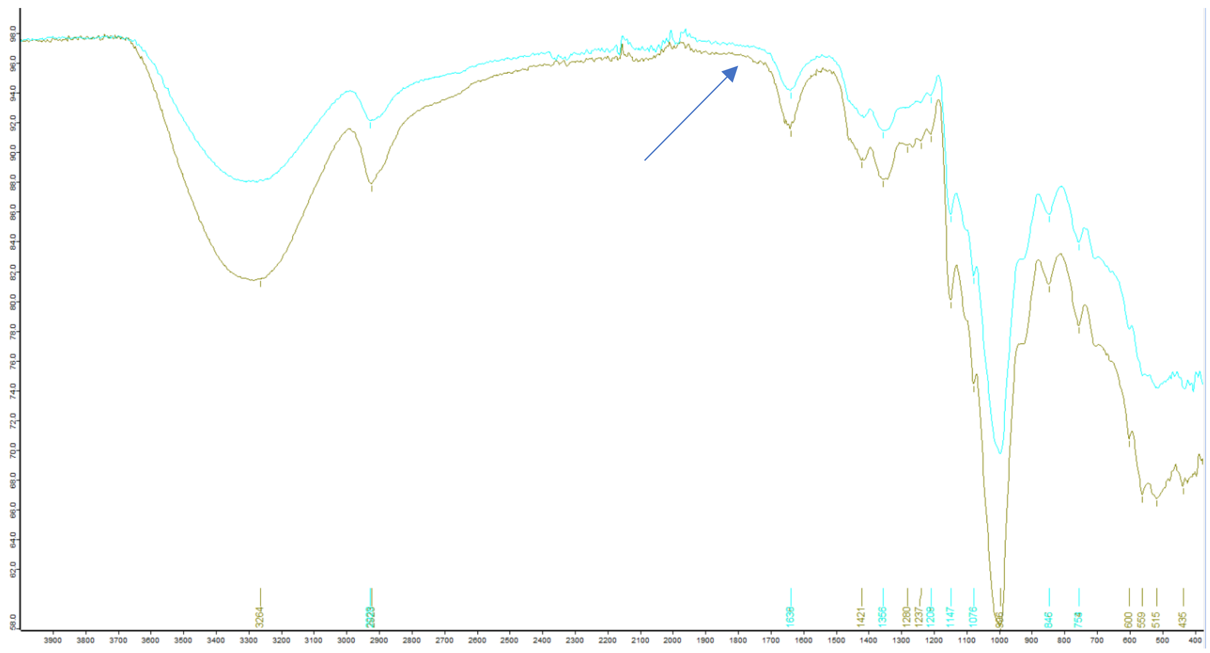

Supp Figure 2: The absence of oil residue in Glycobone® resulting from the precipitation of beads in oil during the crosslinking step, was observed by infrared spectroscopy (MIR) at ~ 1740 cm^{-1}. A typical spectrum of two samples of Glycobone® are depicted. Resolution: 4 cm^{-1}; Scans numbers: 24; Scan between 4000 et 375 cm^{-1}.
