# Supplementary material for: Pullulan–dextran composite beads as bone fillers: from material design and industrial production to clinical application in oral surgery
Source: Front Bioeng Biotechnol. 2026 Jun 4;14:1791131. doi: 10.3389/fbioe.2026.1791131 (PMC13276405; doi:10.3389/fbioe.2026.1791131)
Supplement: Supplementary file 2 [file DataSheet4.pdf]

COMITÉ DE PROTECTION DES PERSONNES
SUD-OUEST ET OUTRE MER III

Président : Docteur Thibaud HAASER
Vice-Présidente : Professeur Marie-Claude SAUX
Secrétaire Générale Adjointe : Madame Marie VIGUIER

NUMERO D'ENREGISTREMENT SI RIPH 2G : 21.03310.000046
NUMERO ID-RCB : 2020-A02457-32

PROMOTEUR : SILTISS

COORDONNATEUR : Docteur Sylvain CATROS

Complément d'information en date du 17 novembre 2021.

En date du 17 novembre 2021, conformément aux dispositions du règlement européen 2017/745 et du code de la Santé Publique, le Comité de Protection des Personnes Sud-Ouest et Outre-mer III a examiné l'investigation clinique portant sur un dispositif médical de classe III, non marqué CE, portant sur la première administration à l'homme ou utilisation chez l'homme d'un produit mentionnée à l'article L. 5311-1 du code de la santé publique, et intitulée :

Étude multicentrique, interventionnelle, longitudinale, ouverte, menée en France, évaluant les performances cliniques et la sécurité de Glycobone, en tant que matériau de comblement osseux dans un contexte de sinus lift d'abord latéral.

Avant de se prononcer, conformément à l'article R. 1123-23 du code de la santé publique, le Comité a formulé une demande de complément d'information et demandé au promoteur de modifier la note d'information.

En date du **15 DECEMBRE 2021**, au vu des réponses et corrections apportées, le Comité à l'unanimité des membres votants émet un

AVIS FAVORABLE

ansm
Agence nationale de sécurité du médicament et des produits de santé

AUTORISATION D'INVESTIGATION CLINIQUE PORTANT SUR UN DISPOSITIF MEDICAL

**Date : 11/01/2022**			
**Identifiants de l'investigation clinique (IC)**			
Titre	Etude multicentrique, interventionnelle, longitudinale, ouverte, menée en France, évaluant les performances cliniques et la sécurité de Glycobone™, en tant que matériau de comblement osseux dans un contexte de sinus lift d'abord latéral.		
Promoteur	SILTISS		
Réf à rappeler		N° IDRCB	**2020-A02457-32**
Catégorie d'IC	*catégorie 2*		

La Directrice générale de l'Agence nationale de sécurité du médicament et des produits de santé (ANSM)

Vu le Règlement (UE) 2017/745 du Parlement européen et du Conseil du 5 avril 2017 relatif aux dispositifs médicaux, modifiant la directive 2001/83/CE, le règlement (CE) n° 174/2002 et le règlement (CE) n° 1223/2009 et abrogeant les directives du Conseil 90/385/CEE et 93/42/CEE, et notamment son article 70.7.b ;

Vu le code de la santé publique et notamment l'article L. 1123-12 ;

Vu le dossier de demande d'autorisation d'investigation clinique adressé à l'ANSM (protocole version 1.1 du 28/10/2021, brochure investigateur version 2 du 27/10/2021);

Vu les compléments versés par le promoteur en date du 03/01/2022 et notamment le protocole de l'essai cité en objet modifié (version 1.3 datée du 03/01/2022), suite à la demande de l'ANSM ;

DECIDE :

L'investigation clinique citée en objet est autorisée.

Supplementary Figure 4: Regulatory French approvals by CPP (Ethical committee - left) and ANSM (Drug and Medical device Agency- right) to start a longitudinal, open-label study to evaluate Glycobone®, as a bone filling material in a sinus lift procedure in 40 patients
