# Supplementary material for: Pullulan–dextran composite beads as bone fillers: from material design and industrial production to clinical application in oral surgery
Source: Front Bioeng Biotechnol. 2026 Jun 4;14:1791131. doi: 10.3389/fbioe.2026.1791131 (PMC13276405; doi:10.3389/fbioe.2026.1791131)
Supplement: Supplementary file 3 [file Table1.docx]

**A**

| **Cell type/Response** | **Score** | | | | |
| --- | --- | --- | --- | --- | --- |
|  | **0** | **1** | **2** | **3** | **4** |
| **Polymorphonuclear cells** | 0 | Rare, 1-5/hpf | 6-10/hpf | Heavy infiltrate | Packed |
| **Lymphocytes** | 0 | Rare, 1-5/hpf | 6-10/hpf | Heavy infiltrate | Packed |
| **Plasma cells** | 0 | Rare, 1-5/hpf | 6-10 /hpf | Heavy infiltrate | Packed |
| **Macrophages** | 0 | Rare, 1-5/hpf | 6-10 /hpf | Heavy infiltrate | Packed |
| **Giant cells/osteoclastic cells** | 0 | Rare, 1-2/hpf | 3-5 /hpf | Heavy infiltrate | Sheets |
| **Necrosis** | 0 | Slight | Moderate | Marked | Severe |
| **Fibrosis/encapsulation** | 0 | Narrowband | Moderately thick band | Thick band | Extensive band with signs of encapsulation |
| **Neovascularization** | 0 | Minimal  capillary proliferation focal  1-3 buds | Groups of 4-7 capillaries with supporting fibroblastic  structures | Broad band of capillaries with supporting structures | Extensive band of capillaries with supporting fibroblastic  Structures |
| **Fatty infiltrate/ Bone marrow** | 0 | Minimal amount of fat associated with fibrosis | Several layers off at and fibrosis | Elongated and broad accumulation of fat cells around the implant site | Extensive fat completely surrounding the implant |

**B**

| **Bone parameters** | **Score** | | | | |
| --- | --- | --- | --- | --- | --- |
|  | 0 | 1 | 2 | 3 | 4 |
| **Schneiderian membrane changes** | 0 | Slight | Moderate | Marked | Severe |
| **Fibrinous exudate (fibrin)** | 0 | Slight | Moderate | Marked | Severe |
| **Osteolysis** | 0 | Slight extent of bone resorption | Moderate extent of bone resorption | Marked extent of bone resorption | Severe extent of bone resorption |
| **Osteoblastic cells** | 0 | Slight = equivalent to normal bone | Moderate  normal bone | Marked  normal bone | Very marked |
| **Bone augmentation** | No bone growth from zygomatic  bone | Bone growth 25% dental implant length | Bone growth 50% dental implant length | Bone growth  75% dental implant length | Bone growth  100% dental implant length |
| **Bone neoformation** | 0 | Slight extent of bone  formation | Moderate extent of bone formation | Marked extent of bone formation | Very marked extent of bone formation |
| **Osteoconduction (biomaterials, implant)** | 0 | Slight | Moderate | Marked | Very marked |
| **Osteointegration**  **biomaterials, implant)** | 0 | Slight | Moderate | Marked | Complete |
| **Bone remodeling** | None (primary  woven bone) | Slight (initial signs of remodeling) | Moderate | Marked | Severe (up to corticalization) |
| **Cortical bone defect repair** | Absence | Slight | Moderate | Marked | Complete |
| **Material degradation** | Absence | Slight | Moderate | Marked | Complete |

**Supplementary Table 1: Histopathological evaluation and scoring systems.** (**A)** Histopathologic evaluation system: inflammatory parameters. These inflammatory parameters are described in the ISO 10993-6 standard, Annex E. hpf: high-power field used in microscopy refers to the field of view under the maximum magnification power of the objective being used (often this represents a 400-fold magnification). (**B)** Histopathologic evaluation system: other tissue response parameters including bone tissue parameters, are added to better characterize the local tissue effects of the two types of biomaterials. These parameters are selected based on previously published systems.
