# Supplementary material for: Pullulan–dextran composite beads as bone fillers: from material design and industrial production to clinical application in oral surgery
Source: Front Bioeng Biotechnol. 2026 Jun 4;14:1791131. doi: 10.3389/fbioe.2026.1791131 (PMC13276405; doi:10.3389/fbioe.2026.1791131)
Supplement: Supplementary file 4 [file DataSheet3.pdf]

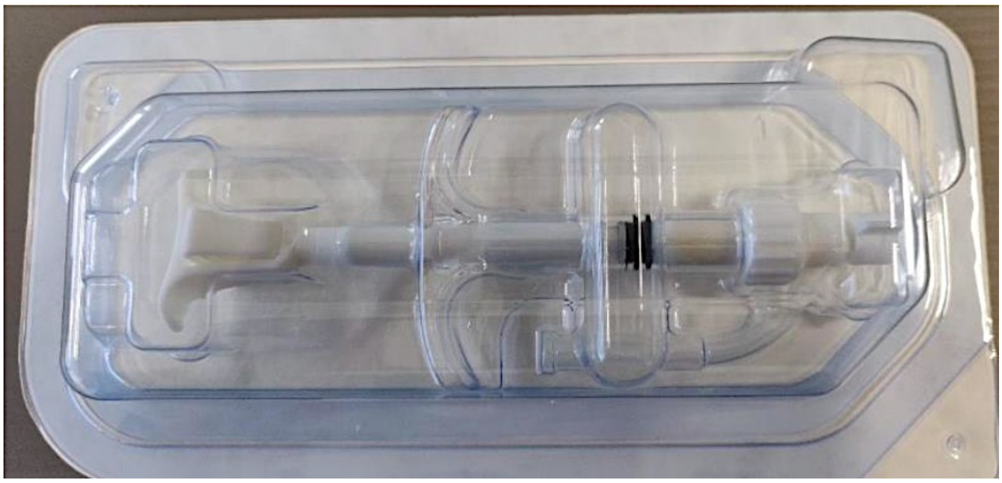

Supplementary Figure 3. Photograph of a syringe filled with Glycobone® beads and a cannula in the final packaging.
