# Supplementary material for: Pullulan–dextran composite beads as bone fillers: from material design and industrial production to clinical application in oral surgery
Source: Front Bioeng Biotechnol. 2026 Jun 4;14:1791131. doi: 10.3389/fbioe.2026.1791131 (PMC13276405; doi:10.3389/fbioe.2026.1791131)
Supplement: Supplementary file 5 [file DataSheet1.pdf]

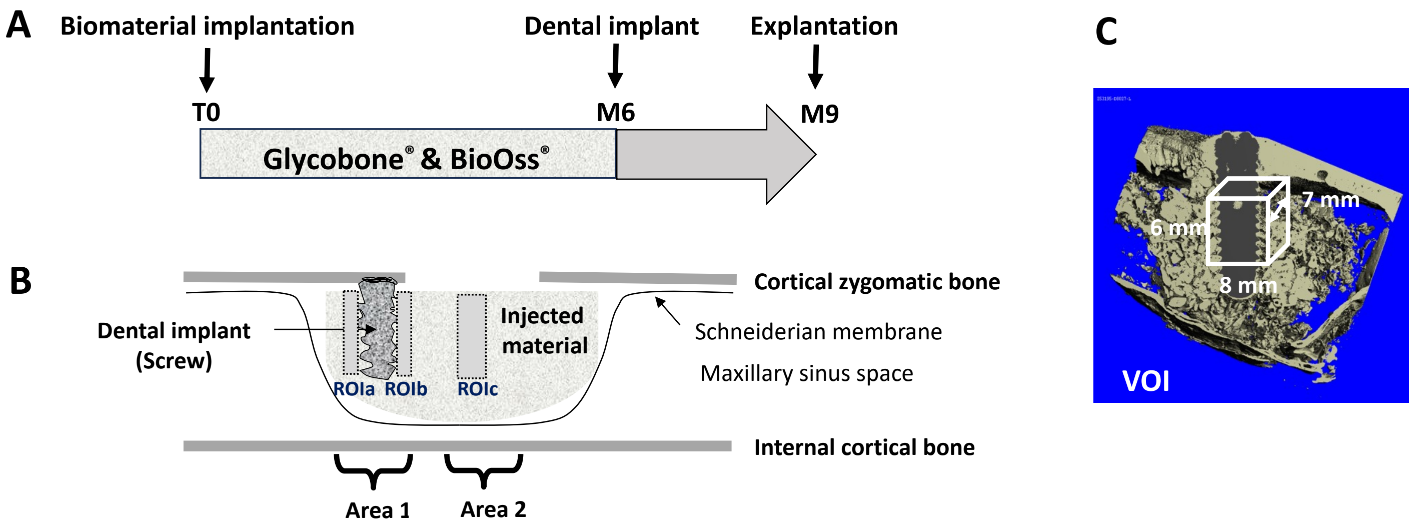

Supplementary Figure 1: (**A) Timeline of implantations**. Both groups of biomaterials (GlycoBone® or BioOss®) were injected within the sinus cavity of sheep (n=6) for 6 months (M6) before the placement of the dental implant. Nine months (M9) after implantation of the biomaterials (three months after the dental implant placement), the sinuses were explanted and treated for micro-CT, histology and histomorphometrical analysis. (**B) Scheme of the region of interest for histological studies**. Two areas were selected: Area 1 included the dental implant for the evaluation of the interface between the dental implant and the injected biomaterials. Area 2 comprised the injected biomaterials, to evaluate the newly formed tissue at distance of the implant. For quantitative analysis of Area 1, two standardized Regions of lnterest (ROI a and ROI b, large of 1000 µm each) were defined as rectangles placed under the cortical bone, on each side of the dental implant, excluding the dental implant. The entire length of the dental implant was considered when defining the ROI a and b. For Area 2: one standardized Region of lnterest ROI c, large of 2000 µm, was defined as a rectangle placed under the cortical bone, at distance from the dental implant. (**C) Scheme of the VOI (Volume of Interest) for Micro-CT quantification** of the mineralized tissue.
